# Impact of a combination of pimobendan, furosemide, and enalapril on heart rate variability in naturally occurring, symptomatic, myxomatous mitral valve degeneration dogs

**DOI:** 10.1186/s12917-023-03770-6

**Published:** 2023-10-12

**Authors:** Prapawadee Pirintr, Nakkawee Saengklub, Pakit Boonpala, Robert L. Hamlin, Anusak Kijtawornrat

**Affiliations:** 1 Department of Veterinary Biosciences and Veterinary Public Health, Faculty of Veterinary Medicine, Chiang Mai University, Chiang Mai, 50100 Thailand; 2 Department of Physiology, Faculty of Pharmacy, Mahidol University, 447 Sriayuthaya Rd., Ratchathewi, Bangkok, 10400 Thailand; 3 Department of Physiology, Faculty of Veterinary Science, Chulalongkorn University, 39 Henri-Dunant Rd., Pathumwan, Bangkok, 10330 Thailand; 4 Department of Veterinary Biosciences, College of Veterinary Medicine, The Ohio State University, 1900 Coffey Rd, Columbus, OH 43210 USA; 5 QTest Labs, LTD, 6456 Fiesta Dr, Columbus, OH 43235 USA; 6 Chulalongkorn University Laboratory Animal Center (CULAC), Chulalongkorn University, Henri-Dunant Rd., Pathumwan, Bangkok 10330, Thailand

**Keywords:** Diuretics, Dogs, Enalapril, Heart rate variability, Mitral valve disease, Pimobendan

## Abstract

**Background:**

Pimobendan, diuretics, and an angiotensin-converting enzyme inhibitor (ACEi) are widely used for the management of chronic valvular heart disease in dogs; however, the effects of that combination on heart rate variability (HRV) are unknown. The purpose of this study was to assess the HRV of symptomatic myxomatous mitral valve degeneration (MMVD) dogs in response to therapy with a combination of pimobendan, diuretics, and ACEi.

**Results:**

MMVD stage C (n = 17) dogs were enrolled and a 1-hour Holter recording together with echocardiography, blood pressure measurement, and blood chemistry profiles were obtained before and 1, 3, and 6 months after oral treatment with pimobendan (0.25 mg/kg), enalapril (0.5 mg/kg), and furosemide (2 mg/kg) twice daily. The results revealed that MMVD stage C dogs at the baseline had lower values of time-domain indices, low frequency (LF), high frequency (HF), and total power, as well as higher value of LF/HF. Triple therapy significantly increases these parameters in MMVD stage C dogs (P < 0.05). A positive moderate correlation was observed between time domain parameters and a left ventricular internal diastole diameter normalized to body weight (P < 0.05).

**Conclusions:**

It can be concluded that MMVD stage C dogs possess low HRV due to either the withdrawal of parasympathetic tone or enhanced sympathetic activation, and a combination therapy was shown to enhance cardiac autonomic modulation inferred from the increased heart rate variability. Therefore, a combination therapy may be useful for restoring normal autonomic nervous system activity in dogs with MMVD stage C.

## Background

Heart rate variability (HRV), fluctuations and oscillations of beat-to-beat RR intervals are surrogate markers for the indirect quantitative assessment of the cardiac autonomic nervous system (ANS), which is a standard method [1]. The alterations in sympathetic and parasympathetic nerve activities to the heart can be assessed from the time and frequency domain of HRV [1]. In human patients with congestive heart failure (CHF), it has been observed that the reduction in HRV is associated with an increased mortality rate [2], as well as sudden cardiac death [3]. In humans and dogs presenting with CHF, the progress of heart disease is related to the degree of cardiac ANS dysfunction, also called sympathovagal imbalance, which gives rise to increased heart rate as well as decreased HRV. In several human studies, higher HRV has been demonstrated as being associated with improved quality of life and reduced mortality [4, 5]. A resent publication has shown that in untreated dogs with MMVD, both sympathetic and parasympathetic tones inferred from HRV parameters were altered before the development of cardiomegaly and prior to clinical signs [6]. Previous publications demonstrated that enalapril and sildenafil improved HRV and could be used to restore sympathovagal balance in dogs with asymptomatic MMVD [7, 8].

Myxomatous mitral valve disease (MMVD) is the most common valvular heart disease in small-to-medium size aging dogs in several parts of the world. Its prevalence increases markedly as dogs become older and it affects males more than females [9]. When the disease progresses, the mitral valve leaks and blood is regurgitated into the left atrium, leading to myocardial remodeling and ventricular dysfunction. Simultaneous with the valve leakage, compensatory mechanisms (i.e., renin angiotensin aldosterone system and sympathetic nervous system) are triggered to compensate for the cardiac function, and dogs are classified as being in the asymptomatic stage. Once dogs experience clinical signs of CHF, furosemide and pimobendan were most prescribed [10]. While angiotensin-converting enzyme inhibitors (ACEi) are controversial since the clinical trials addressing the efficacy of ACEi for the treatment of MMVD dogs have shown mixed outcomes. In 2019, the American College of Veterinary Internal Medicine (ACVIM) consensus guidelines recommended it [9] but the VALVE trial, a newer clinical trial, showed no benefit of using ACEi in MMVD dogs [11]. The new evidence suggested by many clinical trials may impact the future therapy guidelines. In the current study, authors added enalapril, an ACEi, into a combination of pimobendan and furosemide since the ACEi are still widely prescribed based on empirical evidence.

Several drugs used for the treatment of chronic valvular heart disease in dogs have reported their efficacy on survival [12, 13], their effects on heart rate variability are unknown. It is possible that a combination of pimobendan, furosemide, and ACEi may improve sympathetic and parasympathetic tones reflected by changes in HRV. Therefore, this study aimed to evaluate both time and frequency domains of HRV of symptomatic MMVD dogs in response to a combination of those drugs. The clinical significance of this finding will help to better understand the status of sympathovagal balance of dogs with symptomatic MMVD when treated with pimobendan, furosemide, and enalapril.

## Methods

### Inclusion and exclusion criteria

This study was a prospective study, which enrolled client-owned MMVD stage C dogs. The enrolled dogs had to be ≥ 8 years of age which possess MMVD stage C as defined by the ACVIM [9]. The body weight had to be between 4 and 15 kg. Inclusion criteria for the MMVD stage C dogs were mitral regurgitation, cardiac murmur intensity grade *≥* 4/6, a left atrial-to-aortic root ratio *(*LA/Ao*)* ≥ 1.6, a left ventricular internal diastole diameter normalized to body weight *(*LVIDDN*)* ≥ 1.7, and a vertebral heart score > 10.5 [9]. Upon enrollment, each dog had to be newly diagnosed with MMVD stage C and has clinical signs of CHF such as exercise intolerance, coughing, respiratory distress with radiographic evidence of pulmonary congestion and without severe pulmonary edema. The exclusion criteria for all dogs in the study were systemic infection, arrhythmias, pulmonary hypertension *(*tricuspid regurgitation pressure gradient > 65 mmHg*)*, or being treated with other medications related to CHF or medications and/or systemic disorders that have been shown to alter HRV.

### Experimental procedure

Before the beginning of the experiment, a physical examination, echocardiography, electrocardiography (ECG), chest X-ray, blood pressure (BP) examination, hematology, and blood chemistry profiles were performed. After enrollment and baseline (BL) measurement (1 h of Holter recording), the MMVD stage C dogs were given furosemide 2 mg/kg twice per day, enalapril 0.5 mg/kg twice per day, and pimobendan 0.3 mg/kg twice per day. Owners were asked to come back for a follow up 1 (M1), 3 (M3), and 6 (M6) months after treatment for a physical examination and 1 h Holter recording.

### Echocardiography

At baseline, M1, M3, and M6, an echocardiographic examination was performed on all animals without sedation using an ultrasound machine (M9, Mindray Medical, Bangkok, Thailand) equipped with phased array transducers (P10-4E and SP5-1E) by an experienced veterinarian, as previously described [14]. All echocardiographic examinations were performed by a single experienced veterinarian. The left apical 4-chamber view was used to assess the mitral valve and tricuspid valve structures. The continuous-wave Doppler interrogation was used to assess systolic tricuspid regurgitant velocity if tricuspid regurgitation (TR) was identified. The regurgitant velocity was calculated by a modified Bernoulli equation: peak tricuspid regurgitation gradient (TRPG) equal to 4xTRVelocity^2^. The right parasternal short-axis view and M-mode were used to assess the LA/Ao, shortening fraction (SF) and LVIDDN, as previously described [15]. All procedures were performed in accordance with Guidelines for the American Society of Echocardiography [16].

### Thoracic radiography

The right lateral recumbency and ventro-dorsal projection were obtained to visualize the cardiomegaly and pulmonary lesion (i.e., congestion and edema) before the beginning of the study (baseline) and at the end of the study (M6). Cardiomegaly was inferred from the vertebral heart score (VHS), as previously described [17]. The presence of an interstitial or alveolar pattern was used for the evaluation of pulmonary congestion and edema together with clinical signs.

### Electrocardiography

A standard limb lead (i.e., bipolar and unipolar limb leads) electrocardiographic examination was performed while dogs were in a position of right lateral recumbence and all limbs were perpendicular to the body, as previously described [18]. Briefly, four silver–silver chloride ECG electrodes were attached onto the skin of all limbs at the distal to the elbow and knee and connected to an ECG unit (CardiMax FX-8400, Fukuda Denshi, Tokyo, Japan). All tracings of enrolled dogs were analyzed for heart rate and rhythm.

### Blood pressure measurement

An oscillometric unit (petMAP graphic II, CardioCommand, Inc., Tampa, FL, U.S.A.) was used to obtain systolic arterial blood pressure (SBP) at baseline, M1, M3, and M6 by placing the cuff at the right forelimb upon the median artery, between the elbow and the carpal pad. The average consistent blood pressure used for the study was 3, as previously described [19].

### Hematology and blood chemistry profiles

An automated hematology analyzer machine (ProCyte Dx Hematology Analyzer, IDEXX Laboratories, Inc., Westbrook, ME, U.S.A.) was used for CBC collected in the EDTA tube, while a chemistry analyzer machine (IDEXX Catalyst One, IDEXX Laboratories, Inc., Westbrook, ME, U.S.A.) was used for blood chemistry profile measurements collected in a Catalyst sample cup. The hematology (i.e., complete blood count) and blood chemistry profiles (i.e., creatinine; blood urea nitrogen, BUN; alanine aminotransferase, ALT; and alkaline phosphatase, ALKP) were obtained at baseline, M1, M3 and M6.

### Holter recording

At baseline and on the follow-up days (1, 3, and 6 months after treatment), all conscious, resting dogs were subjected to a 1 h continuous Holter recording while they were in a quiet place with their owner, as previously described [20]. Basically, foam monitoring ECG electrodes (3 M™ Red Dot™, 3 M Health Care, St. Paul, MN, USA) were placed on to the skin over the thorax to form transthoracic leads (3 channels) and connected to a 3-channel cardiac Holter monitor Digital Walk (FM-180, Fukuda Denshi Co., Tokyo, Japan). Owners were advised to give drugs to the dogs before 08:00 am and the time for recording was arranged to be performed between 09:00 am and 11:00 am.

### Data analysis

The SCM-510 program (Fukuda Denshi, Tokyo, Japan) was used to analyze all ECG signals obtained from Holter devices and was manually edited by a single experience veterinarian, as previously described [7]. Briefly, an average of the data obtained from 6 to 10 min sections were used in the study. The time and frequency domain parameters of HRV were analyzed using 512 s and a Hamming window. Parameters of the time domain were the average of normal sinus NN durations in the entire recording (NNA); the standard deviation of all normal sinus NN durations in the entire recording (SDNN); the percentage of the number of normal-to-normal sinus NN durations with differences > 50 ms calculated over the entire recording (pNN50); the square root of the average of squared differences between adjacent normal sinus NN durations over the entire recording (rMSSD). The ranges of the frequency domain analysis were set as 0.041–0.15 Hz and 0.15–0.5 Hz for low frequency (LF) and high frequency (HF), respectively. Total power (TP) was calculated from 0 to 0.5 Hz. The estimation of sympathetic and parasympathetic tone relationships (i.e., sympathovagal balance) was calculated as the ratio of LF to HF (LF/HF). The ranges of frequency domain were set in accordance with previous studies in dogs [7, 21, 22].

### Statistical methods

The IBM^®^ SPSS^®^ software platform licensed by Chulalongkorn University was used for the statistical analysis. Percent change from baseline was calculated for HRV parameters. The Shapiro–Wilk test was used for the normality test of the data. Values normally distributed data were shown as mean ± standard deviation (SD). Data among timepoints (i.e., baseline, 1, 3, and 6 months) were compared using one-way repeated measures ANOVA followed by Dunnett’s post hoc test. The Dunnett’s test was used to compare M1, M3, and M6 to a single control timepoint (baseline). If the values failed to display normality, data were shown as median and interquartile range and the Kruskal-Wallis test was used to evaluate the differences between timepoints. Correlations between the HRV parameters and other parameters (i.e., LA/Ao, LVIDDN, SF, VHS, creatinine, BUN, ALT, ALKP) were assessed using the Pearson correlation or Spearman rank correlation in the entire study population. Statistical significance was considered when P-values were less than 0.05.

## Results

A total of 25 client-owned dogs weighing between 4.0 and 15.0 kg were enrolled in the study. Eight dogs were excluded due to client incompliance (n = 3), present of arrhythmias (n = 2), and lost to follow-up (n = 3). Therefore 17 dogs were selected for analysis based on the inclusion criteria. None of the animals in this study had severe congestive heart failure (i.e., pulmonary edema, multiple episodes of syncope) or received any medicine that affected HRV. In addition, all dogs enrolled in this study had no systemic infectious disease that affected heart function as well as HRV. The MMVD dogs had an average age of 12 ± 2.6 years with a median body weight of 5.9 (5–8) kg. The breeds of dogs were Miniature Poodle (n = 6), Shih-Tzu (n = 4), Beagle (n = 2), Cocker Spaniel (n = 1), Schnauzer (n = 1), Yorkshire Terrier (n = 1), Spitz (n = 1), and mix-breed (n = 1). The gender of dogs in this study composed of both male (n = 8) and female (n = 9). Physical examination of all dogs revealed that dogs had a murmur intensity grade IV/VI (n = 10) to V/VI (n = 7). The baseline ECG revealed that the dogs had respiratory sinus arrhythmia and a normal sinus rhythm. In this study, the average dose of pimobendan per meal was 0.28 mg/kg (ranging between 0.25 and 0.31 mg/kg). The average dose of furosemide per meal was 1.84 mg/kg (ranging between 1.67 and 2.22 mg/kg). The average dose of enalapril was 0.51 mg/kg (ranging between 0.42 and 0.62 mg/kg).

**Fig. 1:**
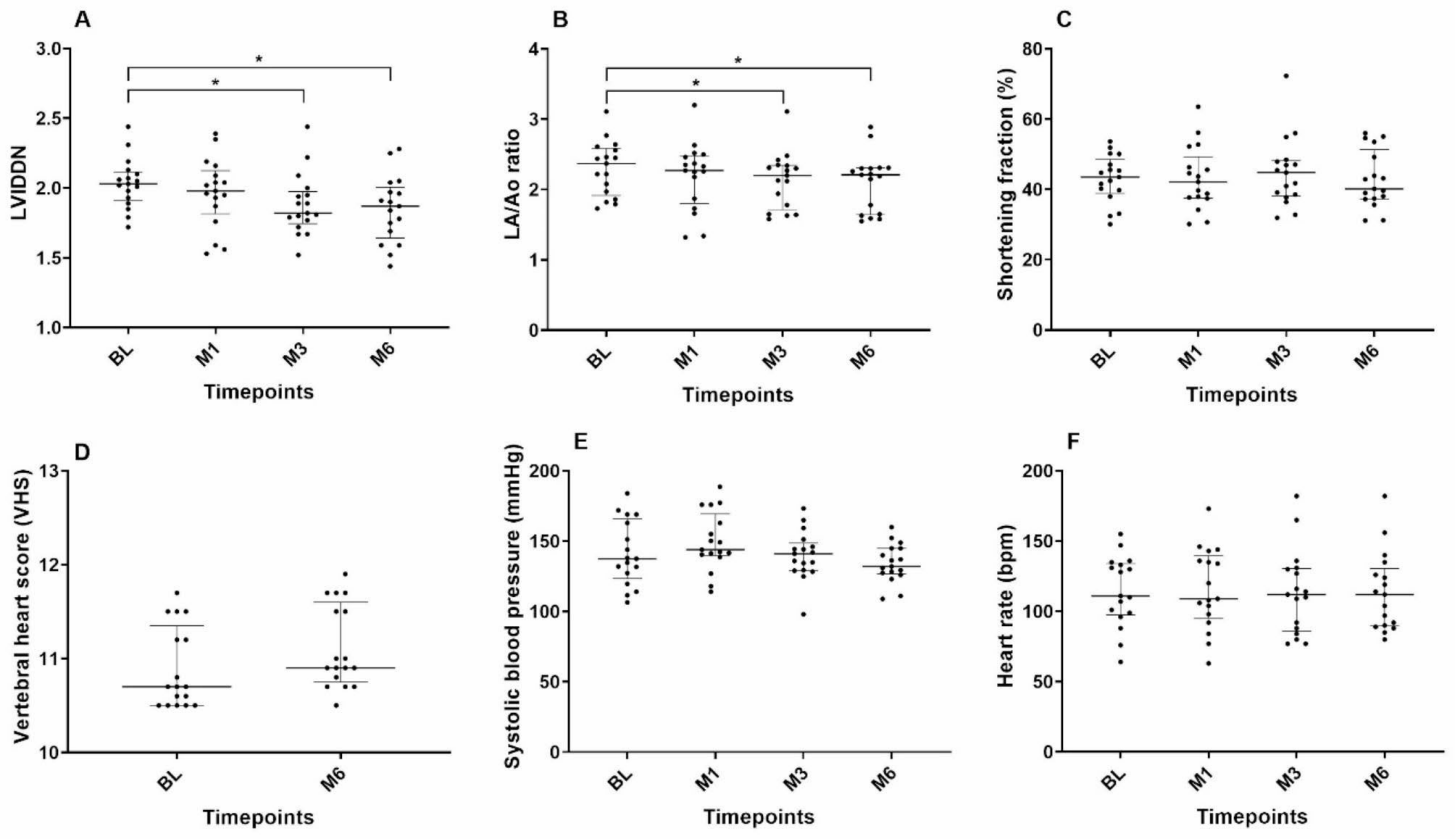
Scatter plots representing the distribution of **(A)** left ventricular internal diastole diameter normalized to body weight (LVIDDN), **(B)** left atrial-to-aortic root ratio (LA/Ao), **(C)** shortening fraction (SF), **(D)** vertebral heart score (VHS), **(E)** systolic blood pressure (SBP), and **(F)** heart rate (HR) including the median and interquartile range of each parameter in dogs with MMVD stage C at the baseline and after treatment with a combination of pimobendan, furosemide, and enalapril at 1 (M1), 3 (M3), and 6 (M6) months; the central horizontal bar in each column represents the median. The error bars represent the 1^st^ and 3^rd^ quartiles. *indicates *P* < 0.05 when compared with baseline value

**Fig. 2:**
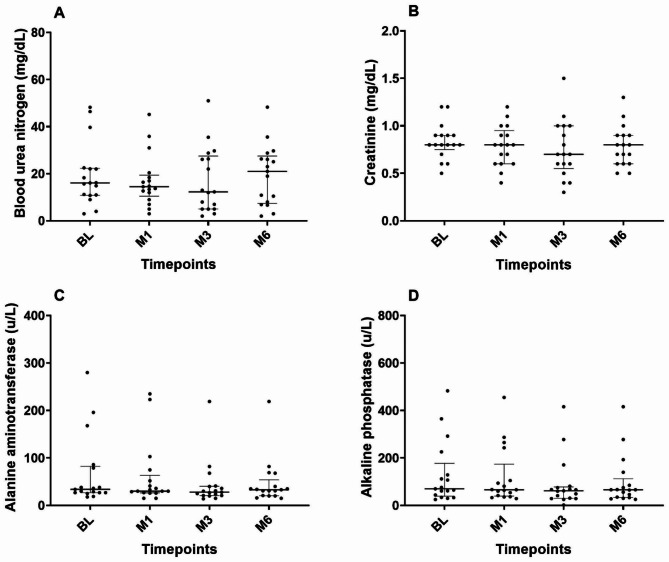
Scatter plots representing the distribution of **(A)** blood urea nitrogen (BUN), **(B)** creatinine, **(C)** alanine aminotransferase (ALT), and **(D)** Alkaline phosphatase (ALKP) including the median and interquartile range of each parameter in dogs with MMVD stage C at the baseline and after treatment with a combination of pimobendan, furosemide, and enalapril at 1 (M1), 3 (M3), and 6 (M6) months; the central horizontal bar in each column represents the median. The error bars represent the 1^st^ and 3^rd^ quartiles

The results of echocardiography, vertebral heart score, systolic blood pressure and blood chemistry profiles were shown in Figs. 1 and 2. The LA/Ao and LVIDDN were declined after treatment and become significantly lower at M3 and M6 when compared with baseline (*P* < 0.05). At baseline, thoracic radiography displayed mild interstitial to alveolar pattern at caudal lobes but not at the 6 months follow up. Vertebral heart score, shortening fraction, systolic blood pressure and heart rate were not different among timepoints (BL vs. M1, BL vs. M3, and BL vs. M6). In addition, all blood chemistry profiles were not altered during treatment when compared with baseline.

**Fig. 3:**
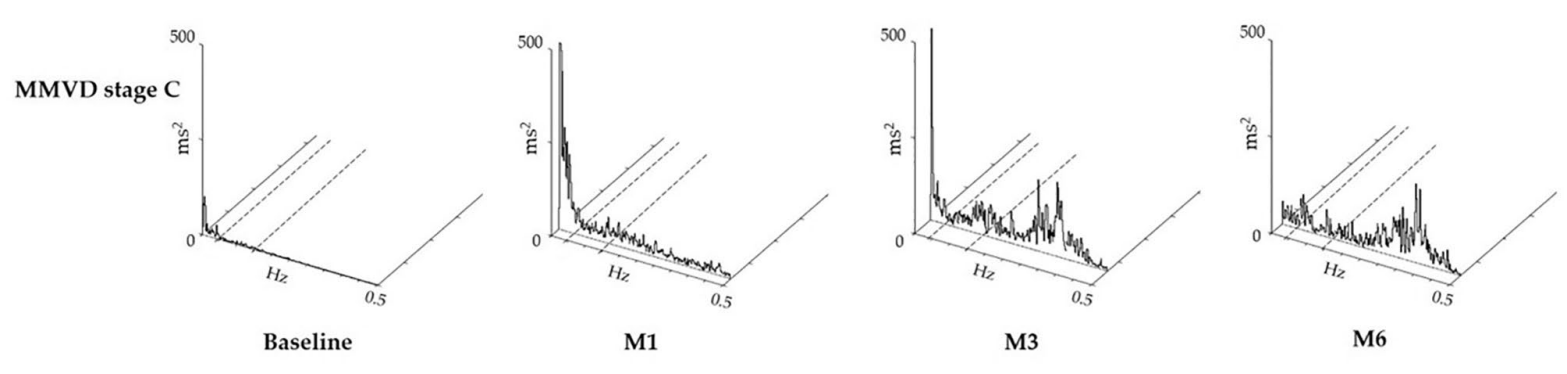
Examples of power spectral density of one dog with MMVD stage C at the baseline and after treatment with a combination of pimobendan, furosemide, and enalapril at 1 **(**M1**)**, 3 **(**M3**)**, and 6 **(**M6**)** months; the Y-bar represents power spectral density at 500 ms^2^ and the X-bar represents a frequency range of 0–0.5 Hz. The dashed line represents the separation between each frequency range **(**i.e., low frequency, 0.041–0.15 Hz; high frequency, 0.15–0.5 Hz**)**

The results of the time and frequency domain analysis of HRV in MMVD stage C dogs at each timepoint is shown in Table 1; Fig. 3. In response to triple therapy, all measured parameters of the time domain were significantly increased at M1, M3, and M6 when compared with the baseline (*P* < 0.05). The percent change of NNA from baseline was increased 13% at M1 and 17% for both M3 and M6. SDNN, rMSSD, and pNN50 were significantly increased at M1, M3 and M6 (SDNN: 57%M1, 76% M3 and 68% M6; rMSSD: 114% M1, 201% M3 and 153% M6; pNN50: 1663% M1, 1729% M3 and 1618% M6).

**Table 1:** Time and frequency domain analysis parameters of heart rate variability in dogs with naturally occurring myxomatous mitral valve degeneration (MMVD) stage C before (baseline) and after treatment with a combination of pimobendan, furosemide, and enalapril assessed at baseline, 1 month (M1), 3 months (M3), and 6 months (M6)

Parameters	MMVD stage C Dogs			
	Baseline	M1	M3	M6
**Time domain**				
**NNA (ms)**	469 ± 99	530 ± 133*	542 ± 130*	542 ± 137*
**SDNN (ms)**	93 ± 42	137 ± 70*	144 ± 68*	143 ± 68*
**rMSSD (ms)**	74 ± 59	138 ± 110*	152 ± 112*	143 ± 110*
**pNN50 (%)**	20 ± 20	38 ± 28*	40 ± 28*	39 ± 27*
**Frequency domain**				
**LF (ms** ^**2**^ **)**	1421 ± 831	2352 ± 2219	2854 ± 2891*	2887 ± 2530*
**HF (ms** ^**2**^ **)**	2597 ± 4980	9986 ± 15,038*	10,703 ± 14,167*	9661 ± 13,701*
**TP (ms** ^**2**^ **)**	7969 ± 7958	19,569 ± 22,171*	20,455 ± 23,302*	20,802 ± 21,413*
**LF/HF**	2.11 ± 2.20	1.12 ± 1.12*	0.90 ± 0.88	1.01 ± 0.91*

Data are shown as mean ± SD, *indicates a significant difference among timepoints (i.e., M1, M3, and M6) when compared to the baseline of each parameter, statistical difference (*P* < 0.05), M month; NNA, the average of normal sinus NN durations in the entire recording; SDNN, standard deviation of all normal sinus NN durations in the entire recording; rMSSD, square root of the average of the squared differences between adjacent normal sinus NN durations over the entire recording; pNN50, the percentage of the number of normal-to-normal sinus NN durations with differences > 50 ms calculated over the entire recording; LF, low frequency; HF, high frequency; TP, total power

The MMVD stage C dogs had increased LF from the first month of treatment and continued to increase and reach statistical significance at M3 (139%) and M6 (642%) when compared with the baseline. The HF of MMVD dogs significantly increased at M1 (881%), M3 (1899%), and M6 (9052%) when compared with the baseline. In response to triple therapy, the TP of MMVD dogs was significantly increased at M1 (213%), M3 (312%), and M6 (1319%), while the LF/HF was significantly decreased at M1 (-33%), M3 (-21%), and M6 (-19%) when compared with the baseline.

The correlation between variables of HRV and indices of echocardiography, VHS, SBP, and blood chemistry profiles was displayed in Table 2. When pooling the data of all timepoints of MMVD stage C dogs, a positive moderate correlation was observed between SDNN and LVIDDN (*P* < 0.001), pNN50 and LVIDDN (*P* < 0.001), and rMSSD and LVIDDN (*P* < 0.01).

**Table 2:** The correlation between variables of heart rate variability and indices of echocardiography, vertebral heart score, systolic blood pressure, and blood chemistry profiles in dogs with naturally occurring myxomatous mitral valve degeneration (MMVD) stage C

Correlation	LF	HF	TP	LF/HF	NNA	SDNN	pNN50%	rMSSD
**LA/Ao**	*r*= -0.097	*r* = 0.083	*r* = 0.081	*r*= -0.125	*r* = 0.282	*r* = 0.322	*r* = 0.319	*r* = 0.317
	*P* = 0.431	*P* = 0.500	*P* = 0.508	*P* = 0.312	*P* = 0.020	*P* = 0.007	*P* = 0.008	*P* = 0.008
**LVIDDN**	*r* = 0.068	*r* = 0.355	*r* = 0.311	*r*= -0.220	*r* = 0.255	*r* = 0.425	*r* = 0.464	*r* = 0.418
	*P* = 0.578	*P* = 0.003	*P* = 0.010	*P* = 0.071	*P* = 0.036	*P* < 0.001	*P* < 0.001	*P* = 0.001
**SF**	*r* = 0.002	*r* = 0.115	*r* = 0.072	*r*= -0.038	*r*= -0.027	*r* = 0.019	*r* = 0.088	*r* = 0.109
(mmHg)	*P* = 0.984	*P* = 0.351	*P* = 0.559	*P* = 0.754	*P* = 0.823	*P* = 0.872	*P* = 0.471	*P* = 0.376
**VHS**	*r= -*0.128	*r= -*0.086	*r= -*0.108	*r =* 0.030	*r= -*0.058	*r= -*0.217	*r= -*0.223	*r= -*0.132
	*P =* 0.469	*P =* 0.626	*P =* 0.542	*P =* 0.865	*P =* 0.741	*P =* 0.217	*P =* 0.204	*P =* 0.456
**SBP**	*r*= -0.166	*r*= -0.250	*r*= -0.242	*r* = 0.102	*r*= -0.238	*r*= -0.354	*r*= -0.290	*r*= -0.257
(mmHg)	*P* = 0.177	*P* = 0.040	*P* = 0.047	*P* = 0.409	*P* = 0.051	*P* = 0.003	*P* = 0.017	*P* = 0.034
**BUN**	*r*= -0.121	*r*= -0.233	*r*= -0.187	*r*= -0.055	*r* = 0.190	*r* = 0.041	*r* = 0.070	*r* = 0.054
(u/L)	*P* = 0.328	*P* = 0.056	*P* = 0.126	*P* = 0.652	*P* = 0.120	*P* = 0.739	*P* = 0.365	*P* = 0.661
**Creatinine**	*r*= -0.083	*r*= -0.190	*r*= -0.160	*r* = 0.067	*r* = 0.166	*r* = 0.019	*r*= -0.011	*r*= -0.047
(u/L)	*P* = 0.498	*P* = 0.120	*P* = 0.192	*P* = 0.584	*P* = 0.175	*P* = 0.874	*P* = 0.930	*P* = 0.704
**ALT**	*r*= -0.182	*r*= -0.220	*r*= -0.220	*r* = 0.039	*r*= -0.204	*r*= -0.303	*r*= -0.276	*r*= -0.238
(mg/dL)	*P* = 0.137	*P* = 0.072	*P* = 0.070	*P* = 0.748	*P* = 0.096	*P* = 0.012	*P* = 0.022	*P* = 0.050
**ALKP**	*r*= -0.138	*r*= -0.220	*r*= -0.230	*r* = 0.054	*r*= -0.119	*r*= -0.323	*r*= -0.333	*r*= -0.332
(mg/dL)	*P* = 0.263	*P* = 0.071	*P* = 0.060	*P* = 0.661	*P* = 0.334	*P* = 0.007	*P* = 0.005	*P* = 0.006

LF, low frequency; HF, high frequency; TP, total power; LF/HF, the ratio of LF to HF; NNA, an average of normal sinus NN durations in the entire recording; SDNN, the standard deviation of all normal sinus NN durations in the entire recording; pNN50, the percentage of the number of normal-to-normal sinus NN durations with differences > 50 ms calculated over the entire recording; rMSSD, the square root of the average of squared differences between adjacent normal sinus NN durations over the entire recording; LA/Ao, a left atrial-to-aortic root ratio; LVIDDN, a left ventricular internal diastole diameter normalized to body weight; SF, shortening fraction; VHS, vertebral heart score; SBP, systolic blood pressure; BUN, blood urea nitrogen; ALT, alanine transaminase; ALKP, alkaline phosphatase

## Discussion

The main goal of this study was to assess the chronic effects of pimobendan, furosemide, and enalapril on time and frequency domain parameters of HRV in naturally occurring, symptomatic MMVD dogs that were freshly diagnosed, without a history of cardiac medication. To achieve this goal, both time and frequency domain analyses was performed from a 1 h continuous ECG recording. As far as we know, this study is the first to report the effects of a combination of pimobendan, furosemide, and enalapril on HRV in dogs with MMVD stage C.

In this study, MMVD dogs were diagnosed through the use of echocardiography, a thoracic radiograph, and cardiac auscultation. In comparison with previous published HRV studies in normal, healthy, age-match control dogs [7, 23], it was found that MMVD dogs had a bigger LA/Ao and LVIDDN than normal healthy dogs. In addition, MMVD dogs also had cardiac murmur intensity and VHS obtained from a thoracic radiograph > 10.5, which was not observed in the normal dogs. This is not surprising because MMVD dogs possess mitral regurgitation due to valvular degeneration [9].

It has been known that the higher the RV: RA pressure gradient the more severity of PAH which will alter the HRV [24]. Therefore, in the current study, MMVD dogs with secondary PAH were excluded. In veterinary medicine, the cutoffs used for PAH categories (i.e., mild, moderate, severe) are arbitrary, and the categories are potentially misleading or flawed [25]. The ACVIM consensus statement guidelines for the diagnosis, classification, treatment, and monitoring of pulmonary hypertension in dogs suggested that the clinical definition of PAH should include dogs with a tricuspid regurgitation PG cutoff of > 46 mmHg (TRV > 3.4 m/s). However, we use the cut-off TRV > 65 mmHg according to the previous publication [15].

It is known that both the sympathetic and parasympathetic nervous systems control cardiac activity; parasympathetic activity is dominant in healthy dogs while the withdrawal of parasympathetic and enhanced sympathetic activities occurs in the presence of heart failure, resulting in reduced HRV [23]. The present study demonstrated that dogs with MMVD stage C had low HRV inferred from the decrease in both time and frequency domain parameters (i.e., SDNN, rMSSD, pNN50, LF, HF, and TP) and an increase in LF/HF. The results from this study are in accordance with previous reports observed in dogs with either symptomatic or asymptomatic MMVD [23, 26–29]. The SDNN, rMSSD, and pNN50 derived from the time domain analysis of HRV were decreased in this study, which is similar to the previous study in MMVD dogs with heart failure [23]. The reductions in SDNN, rMSSD, and pNN50 indicate a reduction in parasympathetic tone [1, 23] and are related to the alteration in frequency domain parameters. In dogs with experimentally induced mild MR, the HF power was significantly decreased while the ratio of LF to HF was significantly increased [26]. Another study of HRV in Cavalier King Charles spaniel dogs with asymptomatic MMVD demonstrated that TP decreased when compared with the control group dogs [29]. The decreasing of HRV in CKCS with HF is also associated with the severity of the disease [27]. The results of decreasing frequency domain parameters in those studies, as well as in our previous study in MMVD stage B1/B2 dogs [7], indicate that dogs with MMVD stage C possess a sympathovagal imbalance, in which there is parasympathetic withdrawal while enhanced sympathetic tones occur.

Previous works by several scholars have reported the restoration of HRV by several pharmacological therapies in MMVD dogs at different stages (e.g., enalapril, sildenafil, and ivabradine) [7, 8, 30]. Short-term use of enalapril was reported as decreasing sympathetic tone while increasing parasympathetic activity in MMVD dogs [8], whereas long-term treatment with enalapril did not show a similar response [7]. Interestingly, long-term treatment with either sildenafil or ivabradine was reported to improve HRV in asymptomatic MMVD dogs [7, 30]. The present study is the first to report the use of a combination of pimobendan, furosemide, and enalapril to restore HRV in dogs with MMVD stage C. An important finding in our study is that chronic treatment with those drugs improves HRV, as indicated by the restoration of sympathovagal balance in MMVD stage C dogs. The increase in parasympathetic tone modulation and improvement of sympathovagal imbalance in MMVD stage C dogs in this study is supported by significantly increased SDNN, rMSSD, and pNN50 of time domain variables and LF, HF, and TP of frequency domain indices of HRV, as well as significantly decreased LF/HF. This finding is also supported by the positive correlations between time domain parameters (i.e., SDNN, rMSSD, pNN50) and progressive reduction of left ventricular internal diastole diameter normalized to body weight. The small but significant reduction in both LA/Ao and LVIDDN after treatment with triple therapy found in the current study is also in accordance with previous studies in dogs with MMVD stage B2 that suggest a decrease in left atrial and ventricular sizes [15, 31].

The effect of either pimobendan or furosemide on HRV has not been studied in dogs; however, the short-term cardiovascular effects mediated by olprinone, a PDE-3 inhibitor, have reportedly increased HRV in adult rabbits [32]. In veterinary medicine, pimobendan, enalapril, and furosemide are widely used for the treatment of congestive heart failure [9]. In dogs with MMVD stage B2, pimobendan has been shown to increase cardiac function, reduce heart size, and prolong the duration of a dog being in stage B2 [31]. In dogs with CHF due to volume overload, pimobendan, angiotensin-converting enzyme inhibitors, and furosemide have demonstrated an ability to restore cardiac function and improve quality of life [33]. Hence, the possible mechanisms of these combination therapies, which improved HRV in this study, might be due to indirect effects on cardiac function, remodeling, and apoptosis as suggested by several scholars [12, 34–37].

This study has certain limitations. Firstly, the number of animals in the study was quite small and the follow up period was only 6 months. However, our results might still be widely applicable for dogs with MMVD stage C as the triple therapy improves sympathovagal balance. A large clinical trial may help to support our findings. Secondly, the current guidelines for the management of MMVD dogs published by ACVIM recommend spironolactone as an adjunct for the chronic treatment of dogs in stage C heart failure to antagonize aldosterone effects [9]. We did not investigate the effect of adding spironolactone to our drug combination on HRV. It has been reported that spironolactone increases HRV in HF patients [38]; therefore, adding spironolactone to our drug combination might increase HRV to a higher level. Further studies with the addition of spironolactone to our drug combination will test this hypothesis. Thirdly, a more resent clinical trial on MMVD dogs has been published, the VALVE trial [11]. This clinical trial demonstrated that addition of ramipril, and ACEi, to pimobendan and furosemide did not have any advantageous outcome on survival time in CHF dogs caused by MMVD. The current study diverges from the VALVE trial in which we tested a combination of drugs (ACEi, diuretic, and pimobendan) instead of pimobendan and diuretics. Therefore, the findings of our study should be interpreted with caution and further clinical trial to assess HRV from MMVD dogs treated with only diuretic and pimobendan should be performed to compare with the current findings. Fourthly, the current study uses 1 h Holter monitoring for analysis of HRV. We are aware that this is not in line with the guideline for HRV measurement, interpretation, and clinical uses published by the European Society of Cardiology and the North American Society of Pacing and Electrophysiology [1]. Therefore, care should be taken when using pathophysiological information from 1 h recording in awake dogs. Nevertheless, from literature, with such variety in procedures of recordings (i.e., timing, parameters, etc.), it remains particularly unclear how long a HRV recording should be to offer the best compromise between the accuracy of the HRV analysis and the comfort for the dogs. In general, the longer the recording time the more reliable the HRV parameters, the shorter the recording the more convenient for the dogs and owners. It has been demonstrated previously by both our groups and others that 1 h of HRV recording and analysis is enough for establishing reliable data if it was performed in the same manner among groups [6–8, 39].

## Conclusions

In conclusion, MMVD stage C dogs possess parasympathetic tone withdrawal and sympathetic tone activation, as indicated by the significant decrease in the indices of the time and frequency domains, as well as the increased LF/HF when compared with healthy control dogs. Symptomatic MMVD stage C dogs treated with a combination of pimobendan, furosemide, and enalapril have increased cardiac autonomic variation. The alteration in HRV parameters in dogs treated with these drugs also indicate the enhancement of parasympathetic tone over sympathetic tone. Therefore, pimobendan, furosemide, and enalapril may be valuable for maintaining normal cardiac autonomic nervous activities in dogs with MMVD stage C.

## Data Availability

The data underlying this article will be shared on reasonable request to the corresponding author.

## Abbreviations

ACVIM: American College of Veterinary Internal Medicine
ALT: alanine aminotransferase
ALKP: alkaline phosphatase
ANS: autonomic nervous system
BUN: blood urea nitrogen
CHF: congestive heart failure
ECG: electrocardiography
HRV: heart rate variability
HF: high frequency
LA/Ao: left atrial-to-aortic root ratio
LVIDDN: left ventricular internal diastole diameter normalized to body weight
LF: low frequency
MMVD: myxomatous mitral valve degeneration
NNA: average of normal sinus NN durations in the entire recording
pNN50: the percentage of the number of normal-to-normal sinus NN durations with differences > 50 ms calculated over the entire recording
rMSSD: square root of the average of squared differences between adjacent normal sinus NN durations over the entire recording
SDNN: standard deviation of all normal sinus NN durations in the entire recording
SBP: systolic arterial blood pressure
TP: total power
VHS: vertebral heart score

## References

[1] Heart rate variability (1996). : standards of measurement, physiological interpretation and clinical use. Task Force of the European Society of Cardiology and the North American Society of Pacing and Electrophysiology. Circulation.

[2] Karcz M, Chojnowska L, Zareba W, Ruzyllo W (2003). Prognostic significance of heart rate variability in dilated cardiomyopathy. Int J Cardiol.

[3] Camm AJ, Pratt CM, Schwartz PJ, Al-Khalidi HR, Spyt MJ, Holroyde MJ (2004). Mortality in patients after a recent myocardial infarction: a randomized, placebo-controlled trial of azimilide using heart rate variability for risk stratification. Circulation.

[4] Sessa F, Anna V, Messina G, Cibelli G, Monda V, Marsala G (2018). Heart rate variability as predictive factor for sudden cardiac death. Aging.

[5] Souza HCD, Philbois SV, Veiga AC, Aguilar BA (2021). Heart Rate Variability and Cardiovascular Fitness: what we know so far. Vasc Health Risk Manag.

[6] Baisan RA, Vulpe V, Ohad DG (2021). Short-term heart rate variability in healthy dogs and dogs in various stages of degenerative mitral valve disease evaluated before pharmacotherapy. Vet J.

[7] Pirintr P, Saengklub N, Limprasutr V, Sawangkoon S, Kijtawornrat A (2017). Sildenafil improves heart rate variability in dogs with asymptomatic myxomatous mitral valve degeneration. J Vet Med Sci.

[8] Chompoosan C, Buranakarl C, Chaiyabutr N, Chansaisakorn W (2014). Decreased sympathetic tone after short-term treatment with enalapril in dogs with mild chronic mitral valve disease. Res Vet Sci.

[9] Keene BW, Atkins CE, Bonagura JD, Fox PR, Haggstrom J, Fuentes VL (2019). ACVIM consensus guidelines for the diagnosis and treatment of myxomatous mitral valve disease in dogs. J Vet Intern Med.

[10] Bode EF, Mederska E, Hodgkiss-Geere H, Radford AD, Singleton DA (2022). Analysis of canine cardiovascular therapeutic agent prescriptions using electronic health records in primary care veterinary practices in the United Kingdom. J Vet Cardiol.

[11] Wess G, Kresken JG, Wendt R, Gaugele J, Killich M, Keller L (2020). Efficacy of adding ramipril (VAsotop) to the combination of furosemide (lasix) and pimobendan (VEtmedin) in dogs with mitral valve degeneration: the VALVE trial. J Vet Intern Med.

[12] Haggstrom J, Boswood A, O’Grady M, Jons O, Smith S, Swift S (2008). Effect of pimobendan or benazepril hydrochloride on survival times in dogs with congestive heart failure caused by naturally occurring myxomatous mitral valve disease: the QUEST study. J Vet Intern Med.

[13] de Madron E, King JN, Strehlau G, White RV (2011). Survival and echocardiographic data in dogs with congestive heart failure caused by mitral valve disease and treated by multiple drugs: a retrospective study of 21 cases. Can Vet J.

[14] Saengklub N, Pirintr P, Nampimoon T, Kijtawornrat A, Chaiyabutr N (2021). Short-term Effects of Sacubitril/valsartan on echocardiographic parameters in Dogs with Symptomatic Myxomatous Mitral Valve Disease. Front Vet Sci.

[15] Boswood A, Haggstrom J, Gordon SG, Wess G, Stepien RL, Oyama MA (2016). Effect of Pimobendan in Dogs with Preclinical Myxomatous Mitral Valve Disease and Cardiomegaly: the EPIC Study-A Randomized Clinical Trial. J Vet Intern Med.

[16] Schiller NB, Shah PM, Crawford M, DeMaria A, Devereux R, Feigenbaum H (1989). Recommendations for quantitation of the left ventricle by two-dimensional echocardiography. American society of Echocardiography Committee on Standards, Subcommittee on quantitation of Two-Dimensional Echocardiograms. J Am Soc Echocardiogr.

[17] Buchanan JW, Bucheler J (1995). Vertebral scale system to measure canine heart size in radiographs. J Am Vet Med Assoc.

[18] Tilley LP, Tilley LP (1992). Essentials of Canine and Feline Electrocardiography: interpretation and treatment. Principles of electrocardiographic recording.

[19] Acierno MJ, Brown S, Coleman AE, Jepson RE, Papich M, Stepien RL (2018). ACVIM consensus statement: guidelines for the identification, evaluation, and management of systemic hypertension in dogs and cats. J Vet Intern Med.

[20] Petrie JP (2005). Practical application of holter monitoring in dogs and cats. Clin Tech Small Anim Pract.

[21] Dong VNK, Tantisuwat L, Setthawong P, Tharasanit T, Sutayatram S, Kijtawornrat A (2022). The preliminary Chronic Effects of Electromagnetic Radiation from Mobile Phones on Heart Rate Variability, Cardiac function, blood profiles, and Semen Quality in Healthy Dogs. Vet Sci.

[22] Pirintr P, Chansaisakorn W, Trisiriroj M, Kalandakanond-Thongsong S, Buranakarl C (2012). Heart rate variability and plasma norepinephrine concentration in diabetic dogs at rest. Vet Res Commun.

[23] Oliveira MS, Muzzi RA, Araujo RB, Muzzi LA, Ferreira DF, Nogueira R (2012). Heart rate variability parameters of myxomatous mitral valve disease in dogs with and without heart failure obtained using 24-hour Holter electrocardiography. Vet Rec.

[24] Carvalho CG, Bresler R, Zhi YX, Alshaer H, Granton JT, Ryan CM (2019). Heart rate variability in pulmonary hypertension with and without sleep apnea. Heliyon.

[25] Reinero C, Visser LC, Kellihan HB, Masseau I, Rozanski E, Clercx C (2020). ACVIM consensus statement guidelines for the diagnosis, classification, treatment, and monitoring of pulmonary hypertension in dogs. J Vet Intern Med.

[26] Fujii Y, Wakao Y (2003). Spectral analysis of heart rate variability in dogs with mild mitral regurgitation. Am J Vet Res.

[27] Haggstrom J, Hamlin RL, Hansson K, Kvart C (1996). Heart rate variability in relation to severity of mitral regurgitation in Cavalier King Charles spaniels. J Small Anim Pract.

[28] Oliveira MS, Muzzi RAL, Araújo RB, Muzzi LAL, Ferreira DF, Silva EF (2014). Heart rate variability and arrhythmias evaluated with Holter in dogs with degenerative mitral valve disease. Arq Bras Med Vet Zootec.

[29] Rasmussen CE, Falk T, Zois NE, Moesgaard SG, Haggstrom J, Pedersen HD (2012). Heart rate, heart rate variability, and arrhythmias in dogs with myxomatous mitral valve disease. J Vet Intern Med.

[30] Pirintr P, Limprasutr V, Saengklub N, Pavinadol P, Yapao N, Limvanicharat N (2018). Acute effect of ivabradine on heart rate and myocardial oxygen consumption in dogs with asymptomatic mitral valve degeneration. Exp Anim.

[31] Boswood A, Gordon SG, Haggstrom J, Wess G, Stepien RL, Oyama MA (2018). Longitudinal analysis of Quality of Life, Clinical, Radiographic, echocardiographic, and laboratory variables in Dogs with Preclinical Myxomatous Mitral Valve Disease receiving Pimobendan or Placebo: the EPIC Study. J Vet Intern Med.

[32] Mokra D, Tonhajzerova I, Pistekova H, Visnovcova Z, Mokry J, Drgova A (2013). Short-term cardiovascular effects of selective phosphodiesterase 3 inhibitor olprinone versus non-selective phosphodiesterase inhibitor aminophylline in a meconium-induced acute lung injury. J Physiol Pharmacol.

[33] Atkins C, Bonagura J, Ettinger S, Fox P, Gordon S, Haggstrom J (2009). Guidelines for the diagnosis and treatment of canine chronic valvular heart disease. J Vet Intern Med.

[34] Bagardi M, Zamboni V, Locatelli C, Galizzi A, Ghilardi S, Brambilla PG. Management of chronic congestive heart failure caused by Myxomatous Mitral Valve Disease in Dogs: a narrative review from 1970 to 2020. Anim (Basel). 2022;12(2). 10.3390/ani12020209 PMC8773235 35049831

[35] Group TES (2002). Effects of pimobendan on adverse cardiac events and physical activities in patients with mild to moderate chronic heart failure: the effects of pimobendan on chronic heart failure study (EPOCH study). Circ J.

[36] Lombard CW, Jons O, Bussadori CM (2006). Clinical efficacy of pimobendan versus benazepril for the treatment of acquired atrioventricular valvular disease in dogs. J Am Anim Hosp Assoc.

[37] Nonaka M, Morimoto S, Murayama T, Kurebayashi N, Li L, Wang YY (2015). Stage-dependent benefits and risks of pimobendan in mice with genetic dilated cardiomyopathy and progressive heart failure. Br J Pharmacol.

[38] Tacoy G, Balcioglu AS, Arslan U, Durakoglugil E, Erdem G, Ozdemir M (2007). Effect of metoprolol on heart rate variability in symptomatic patients with mitral valve prolapse. Am J Cardiol.

[39] Bogucki S, Noszczyk-Nowak A (2017). Short-term heart rate variability in dogs with sick sinus syndrome or chronic mitral valve disease as compared to healthy controls. Pol J Vet Sci.

